# Association between maternal smoking during pregnancy and learning disabilities in children and adolescents: A propensity score matching analysis

**DOI:** 10.18332/tid/214128

**Published:** 2025-12-23

**Authors:** Pingping Li, Tong Lu, Wei Wang, Linjun Du

**Affiliations:** 1 Graduate School of Zhejiang Chinese Medical University, Hangzhou, China; 2 Department of Child Healthcare, Yueqing Maternal and Child Health Hospital, Wenzhou, China; 3 Emergency Department, Liaocheng Maternal and Child Health Hospital, Liaocheng, China; 4 Department of Child Healthcare, Tai'an Maternal and Child Health Hospital, Tai'an, China; 5 Department of Child Healthcare, The Third People's Hospital of Liaocheng City, Liaocheng, China

**Keywords:** LDs, maternal smoking during pregnancy, children and adolescents, propensity score matching

## Abstract

**INTRODUCTION:**

The evidence on the associations between maternal smoking during pregnancy and long-term neurodevelopmental outcomes in offspring, particularly learning disabilities, remains insufficient. This study aimed to evaluate the association between maternal smoking during pregnancy and learning disabilities (LDs) in children and adolescents.

**METHODS:**

This cross-sectional study used data from the National Health and Nutrition Examination Survey (NHANES) cycles 1999–2004. Maternal smoking status during pregnancy was obtained from self-reported questionnaires and classified as smoking or non-smoking. The primary outcome, learning disabilities (LDs), was determined based on parental response to the question: ‘Has a doctor or other health professional ever told you that your child has a learning disability?’. Multiple analytic techniques, including multivariable logistic regression, propensity score matching (PSM), doubly robust estimation, inverse probability weighting (IPW), standardized mortality ratio weighting (SMRW), and stratified analyses, were used to evaluate the robustness of our findings.

**RESULTS:**

There were 5835 participants in all, of whom 848 had mothers who smoked during pregnancy and 4987 had mothers who did not. The prevalence of LD was 18.9% (160/848) in the smoking group compared with 9.5% (474/4987) in the non-smoking group. After PSM, 1666 matched individuals were identified. The IPW model indicated that maternal smoking during pregnancy was significantly associated with LDs in offspring (AOR=1.94; 95% CI: 1.59–2.37). Consistent results were confirmed by multivariable logistic regression, doubly robust estimation, SMRW, and stratified analyses.

**CONCLUSIONS:**

Maternal smoking during pregnancy was positively associated with LDs among US children and adolescents. It is necessary to conduct further prospective studies to better understand this relationship.

## INTRODUCTION

LDs are a category of neurodevelopmental disorders characterized by difficulties in understanding new or complex information and acquiring academic skills such as reading, writing, and mathematics, despite normal intelligence and educational opportunities^1^. According to the latest data brief released by the US National Center for Health Statistics (NCHS) in 2022, 8.76% of US children and adolescents were diagnosed with LD between 1997 and 2021^2^. This condition is one of the primary causes of poor academic performance and psychosocial issues in adulthood. Identifying modifiable risk factors, particularly those related to maternal exposures during pregnancy – such as smoking, alcohol use, and nutritional status – is of significant public health importance, as early prevention and intervention may improve long-term cognitive development, educational outcomes, and social adaptation^3^.

Pregnancy-related maternal smoking is still a significant global public health concern^4^. According to data from the Centers for Disease Control and Prevention in 2023, 4.6% of pregnant women still smoke^5^, despite a reduction in smoking rates over the past few decades^6^. Numerous negative effects, including low birth weight, preterm birth, stillbirth, and sudden infant death syndrome, have been connected to prenatal exposure to tobacco smoking^4,7,8^. Additionally, there is growing evidence that maternal smoking may have long-term impacts on the neurodevelopment of offspring, including behavioral issues, cognitive impairment^9-11^, and attention-deficit/hyperactivity disorder (ADHD)^12,13^.

The neurotoxic effects of carbon monoxide and nicotine decrease placental blood flow^11^, and epigenetic changes like DNA methylation that may have long-lasting impacts on brain development are some of the molecular pathways that may underlie these relationships^14^. The link between mothers smoking during pregnancy and learning problems in children and adolescents is still poorly understood, despite the evidence that supports it. Small sample sizes, insufficient confounding factor adjustment, or a failure to use sophisticated statistical techniques to address selection bias have frequently been the limitations of prior research. To ascertain whether mother smoking on its own increases the likelihood of learning problems in offspring, extensive population studies using exacting analytical techniques are required.

Thus, this study examines the relationship between maternal smoking during pregnancy and learning problems in children and adolescents using data from the NHANES. As per the hypothesis of the study, maternal smoking during pregnancy is associated with an increased likelihood of LDs in children and adolescents.

## METHODS

### Data acquisition and ethics statement

**Figure 1:**
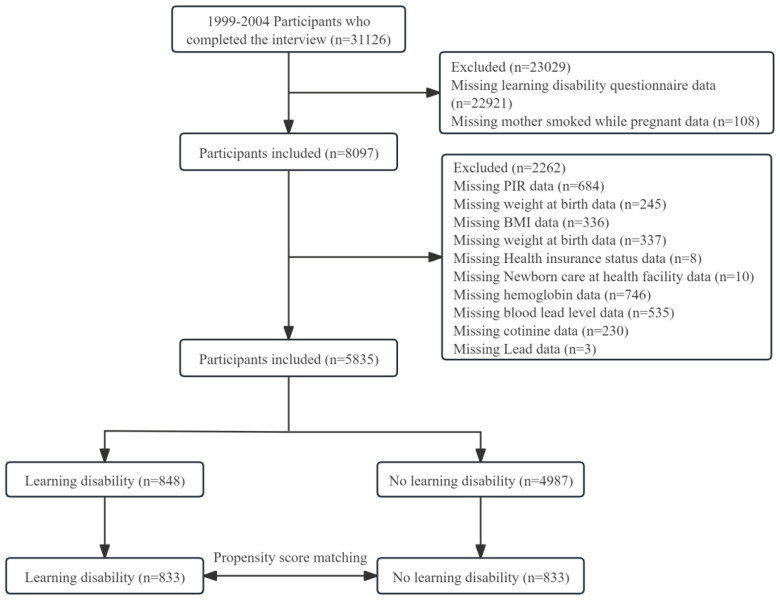
Study flow diagram

This study analyzed data collected from each cycle of the National Health and Nutrition Examination Survey (NHANES) in the United States from 1999 to 2004^15^, with data on learning difficulties restricted to children and adolescents between the ages of 4 and 15 years^15^. In order to guarantee national representation, the National Center for Health Statistics (NCHS) uses a stratified multistage cluster sampling technique that is based on probability. Home visits were used to gather demographic and health data, and then Mobile Examination Centers (MECs) were used for physical examinations and assessments^16^. The Institutional Review Board (IRB) of NCHS gave its approval for this study. Prior to inclusion, all subjects gave written informed consent; further IRB approval was not needed for secondary analyses^5^. Participants having incomplete information on confounders, learning disability questionnaire answers, or mother smoking during pregnancy were not included in this study, which concentrated on children and adolescents ages 4 to 15 years. Figure 1 illustrates the participant selection process, resulting in 5835 participants being included in the final analysis. This study adheres to the strengthened Declaration on the Reporting of Observational Studies in Epidemiology and the Declaration of Helsinki^17,18^.

### Maternal smoking during pregnancy

Responses to the questionnaire question: ‘Did your birth mother smoke at any time during her pregnancy?’ were used to gauge maternal smoking during pregnancy. All other answers were defined as non-smoking; however, a ‘yes’ response was considered smoking^19,20^.

### LD

Parents’ answer to the question: ‘Has a school representative or health professional ever told you that your child has a learning disability?’ is used to identify learning disability. If the answer was ‘yes’, the child had a learning disability; if the answer was ‘no’, the child was classified as not having one^21,22^.

### Covariates

Sex, age, BMI, race/ethnicity, poverty income ratio (PIR), household size, place of birth, health insurance status, birth weight, cotinine, hemoglobin, and blood lead were among the variables we collected from NHANES based on previous research^12,20,21,23-25^. Hispanic/Latino, other Hispanic, non-Hispanic White, non-Hispanic Black, and Other races were the specific race/ethnicity categories^26^. PIR was computed by taking the survey year’s poverty threshold and dividing it by household (or individual) income^27^. Household income was divided into three PIR classes based on a US government report: low (PIR≤1.3), moderate (PIR=1.3–3.5), and high (PIR>3.5). There are two categories for household size: ≤4 and >4. Using isotope dilution-high-performance liquid chromatography/atmospheric pressure chemical ionization tandem mass spectrometry (ID HPLC-APCI MS/MS), the amount of serum cotinine (ng/mL) in NHANES was measured. Every participant’s hemoglobin (HGB, g/dL) was measured with a Beckman Coulter DxH 800 device. A PerkinElmer SIMAA 6000 synchronous multi-element atomic absorption spectrometer with Zeeman background correction was used to measure the levels of lead in blood (umol/L). All of the variable data are available on the NHANES website (https://wwwn.cdc.gov/nchs/nhanes/Default.aspx).

### Statistical analysis

For descriptive analysis, participants were split into two groups according to the mother’s smoking status during pregnancy. Continuous variables with a normal distribution are shown as mean ± standard deviation (SD). The median and interquartile range (IQR) are used to express data that are not regularly distributed. When reporting categorical variables, percentages and frequencies are utilized. Fisher’s exact tests or chi-squared tests were used to assess categorical data, while t-tests or one-way ANOVA were used to compare baseline characteristics between groups for continuous variables.

To minimize potential bias, logistic regression analysis and propensity score matching (PSM) were used to balance confounding factors between groups. Sex, age, race/ethnicity, BMI, PIR, household size, healthcare institution for neonatal care, health insurance status, birth weight, cotinine, hemoglobin, and blood lead levels were among the matching variables included in PSM. A 10% standard deviation was thought to be adequate to balance distributions, and participants were matched between groups using a matching caliper (0.2). In addition to the odds ratio (OR) and 95% CI for each estimate, propensity scores were computed using logistic regression models.

The dual robustness assessment method combines multivariate regression models with propensity score matching (PSM) to estimate the association between exposure and outcome, potentially yielding unbiased effect estimates. Therefore, we employed this dual robustness assessment method to further confirm the association between maternal smoking during pregnancy and LD. R software (version 4.0.0) and Free Statistics software (version 2.2) were used to conduct the statistical analyses in this study. A bilateral p<0.05 was deemed statistically significant.

## RESULTS

### Population and baseline characteristics

There were 31126 participants in the 1999–2004 NHANES database. In the final analysis, we included 5835 children and adolescents (aged 4–15 years) after excluding cases with incomplete information on confounders, learning disability questionnaires, and maternal smoking during pregnancy. Of them, 4987 (85.5%) had birth mothers who did not smoke throughout pregnancy, and 848 (14.5%) had mothers who smoked during pregnancy. Eight hundred thirty-three matched pairs with balanced patient characteristics were found via propensity score matching (Figure 1).

Table 1 displays the baseline attributes of each participant; 2546 (51.1%) of the participants were female, and their average age was 10.35 years. Prior to PSM, the two groups’ differences in gender, age, hemoglobin levels, and learning difficulties were statistically significant. Eight hundred thirty-three couples were matched after PSM. The standardized differences of variables between the mother smoking during pregnancy group and the non-smoking group were <10%, with the exception of race/ethnicity and PIR.

**Table 1:** Demographics and baseline characteristics of patients before and after propensity score matching, National Health and Nutrition Examination Survey (NHANES) cycles 1999–2004

*Characteristics*	*Unmatched patients*			*Propensity score matched patients*		
	*Mother did not* *smoke while* *pregnant* *(N=4987)* *n (%)*	*Mother smoked* *while pregnant* *(N=848)* *n (%)*	*SMD*	*Mother did not* *smoke while* *pregnant* *(N=833)* *n (%)*	*Mother smoked* *while pregnant* *(N=833)* *n (%)*	*SMD*
**Sex**			0.035			0.005
Male	2441 (48.9)	430 (50.7)		418 (50.2)	420 (50.4)	
Female	2546 (51.1)	418 (49.3)		415 (49.8)	413 (49.6)	
**Age** (years), mean (SD)	10.35 (3.44)	10.50 (3.43)	0.042	10.41 (3.40)	10.45 (3.41)	0.013
**Race/ethnicity**			0.551			0.126
Mexican American	1830 (36.7)	136 (16.0)		154 (18.5)	135 (16.2)	
Other Hispanic	223 (4.5)	28 (3.3)		20 (2.4)	28 (3.4)	
Non-Hispanic White	1194 (23.9)	366 (43.2)		367 (44.1)	353 (42.4)	
Non-Hispanic Black	1553 (31.1)	271 (32.0)		234 (28.1)	270 (32.4)	
Other/Multiracial	187 (3.7)	47 (5.5)		58 (7.0)	47 (5.6)	
**BMI** (kg/m^2^), mean (SD)	20.30 (5.22)	20.78 (5.56)	0.09	20.87 (5.78)	20.69 (5.41)	0.032
**PIR**			0.13			0.053
Low	2226 (44.6)	417 (49.2)		390 (46.8)	408 (49.0)	
Medium	1776 (35.6)	303 (35.7)		321 (38.5)	300 (36.0)	
High	985 (19.8)	128 (15.1)		122 (14.6)	125 (15.0)	
**Household size**			0.089			<0.001
≤4	2255 (45.2)	421 (49.6)		411 (49.7)	411 (49.7)	
>4	2732 (54.8)	427 (50.4)		422 (50.7)	422 (50.7)	
**Newborn care at health facility**			0.113			<0.001
No	4419 (88.6)	719 (84.8)		707 (84.9)	707 (84.9)	
Yes	568 (11.4)	129 (15.2)		126 (15.1)	126 (15.1)	
**Health insurance status**			0.093			0.011
Not insured	850 (17.0)	116 (13.7)		111 (13.3)	114 (13.7)	
Insured	4137 (83.0)	732 (86.3)		722 (86.7)	719 (86.3)	
**Weight at birth** (pounds), mean (SD)	6.91 (1.38)	6.52 (1.48)	0.272	6.54 (1.53)	6.52 (1.45)	0.015
**Cotinine** (ng/mL), mean (SD)	1.56 (13.18)	7.88 (34.07)	0.245	3.86 (21.76)	4.70 (18.06)	0.042
**Hemoglobin** (g/dL), mean (SD)	13.48 (1.11)	13.51 (1.12)	0.026	13.53 (1.06)	13.49 (1.11)	0.041
**Lead** (umol/L), mean (SD)	1.62 (1.28)	1.83 (1.61)	0.145	1.78 (1.60)	1.82 (1.56)	0.027
**Learning disabilities**			<0.001			<0.001
No	4513 (90.5)	688 (81.1)		730 (87.6)	679 (81.5)	
Yes	474 (9.5)	160 (18.9)		103 (12.4)	153 (18.5)	

PIR: ratio of income to poverty. BMI: body mass index. SMD: standardized mean difference.

### Association between maternal smoking during pregnancy and learning disabilities

**Figure 2:**
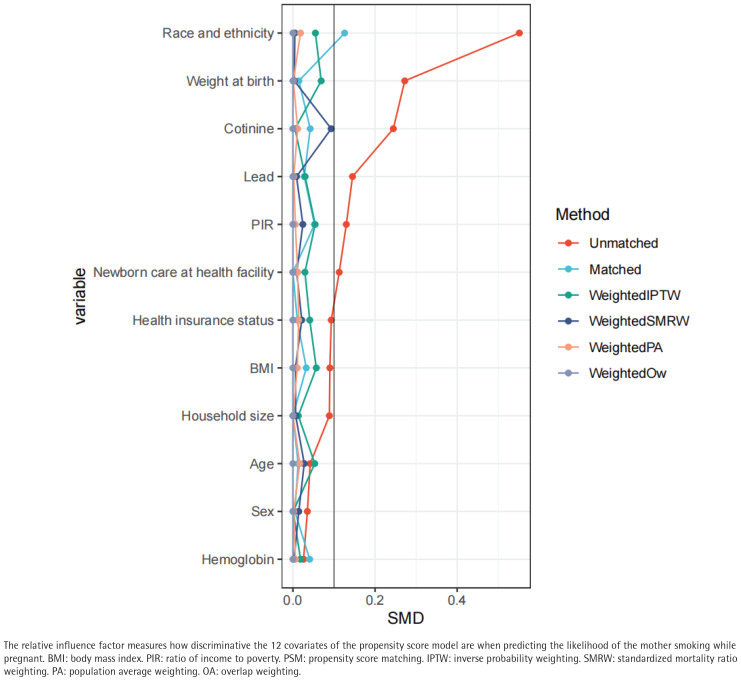
Relative influence factor of covariates, related to predicting the likelihood of the mother smoking while pregnant, National Health and Nutrition Examination Survey (NHANES) cycles 1999–2004

The propensity score model was first developed using these 12 factors, and Figure 2 shows how each factor contributed to the final propensity score. Standardizing the discrepancies between pregnant mothers who smoked and those who did not was done using inverse probability weighting, based on the calculated propensity scores. Except for race/ethnicity and PIR, the majority of factors were ‘equal’ or fairly balanced between the smoking and non-smoking groups, as seen in Table 1.

**Figure 3:**
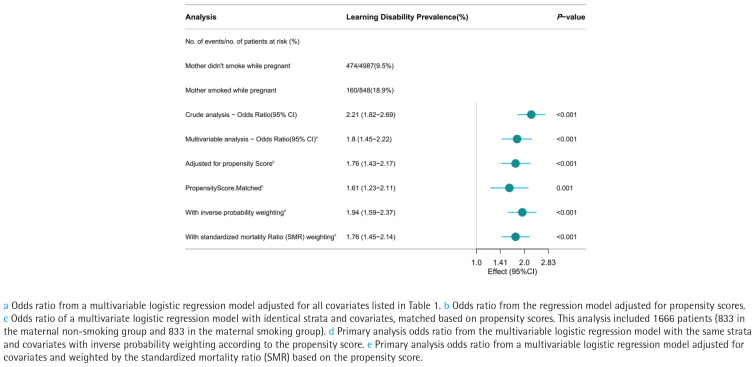
The forest plot displays the odds ratios (ORs) for the prevalence of learning disabilities using various models within the National Health and Nutrition Examination Survey (NHANES) cycles 1999–2004

Overall, 10.9% of people had learning problems (634/5835). In the mother smoking group, the prevalence of learning difficulties was 18.9% (160/848), while in the maternal non-smoking group, it was 9.5% (474/4987) (Figure 3). LD had higher odds in the maternal smoking group than in the non-smoking group in the unadjusted model (OR=2.21; 95% CI: 1.82–2.69, p<0.001). The logistic regression’s OR for mother smoking during pregnancy, after controlling for covariates, was 1.8 (95% CI: 1.45–2.22, p<0.001). Among the 1666 matched participants, the odds ratio adjusted for propensity score was 1.76 (95% CI: 1.43–2.17, p<0.001). In a multivariate logistic regression model based on propensity score matching with identical stratification and covariates, the OR was 1.61 (95% CI: 1.23–2.11, p=0.001). Both IPTW (OR=1.94; 95% CI: 1.59–2.37, p<0.001) and SMRW (OR=1.76, 95% CI: 1.45–2.14, p<0.001) analyses demonstrated a positive association between maternal smoking during pregnancy and the prevalence of learning disabilities (Figure 3).

We performed a dual robustness analysis on the PSM-adjusted data, namely a multi-model adjustment for logistic regression, as indicated in Table 2. Age, sex, race/ethnicity, BMI, and PIR were all taken into account in Model 1. Model 2 was adjusted as for Model 1 plus birth weight, neonatal care at hospitals, and health insurance status. Model 3 was adjusted as for Model 2 plus hemoglobin, cotinine, and blood lead levels. With an AOR of 1.6 (95% CI: 1.21–2.12; p=0.001), compared to the non-smoking group, children and adolescents in the current pregnancy smoking group face a higher likelihood of reporting learning difficulties. This further shows that the prevalence of learning problems and maternal smoking during pregnancy are independently correlated (Table 2).

**Table 2:** Post-matched multivariable regression of mother smoked while pregnant and learning disabilities, National Health and Nutrition Examination Survey (NHANES) cycles 1999–2004

*Mother* *smoked* *while* *pregnant*	*Total*	*n (%)*	*Unadjusted* *OR (95% CI)*	*p*	*Model 1* *AOR (95% CI)*	*p*	*Model 2* *AOR (95% CI)*	*p*	*Model 3* *AOR (95% CI)*	*p*
No (ref.)	833	103 (12.4)	1		1		1		1	
Yes	833	154 (18.5)	1.61 (1.23–2.11)	0.001	1.6 (1.21–2.11)	0.001	1.61 (1.22–2.13)	0.001	1.6 (1.21–2.12)	0.001

AOR: adjusted odds ratio. Model 1: adjusted for age, sex, race/ethnicity, BMI and PIR. Model 2: adjusted as for Model 1 plus weight at birth, newborn care at health facility, health insurance status, and household size. Model 3: adjusted as for Model 2 plus hemoglobin, cotinine, and lead. PIR: ratio of income to poverty. BMI: body mass index.

### Subgroup analysis

**Figure 4:**
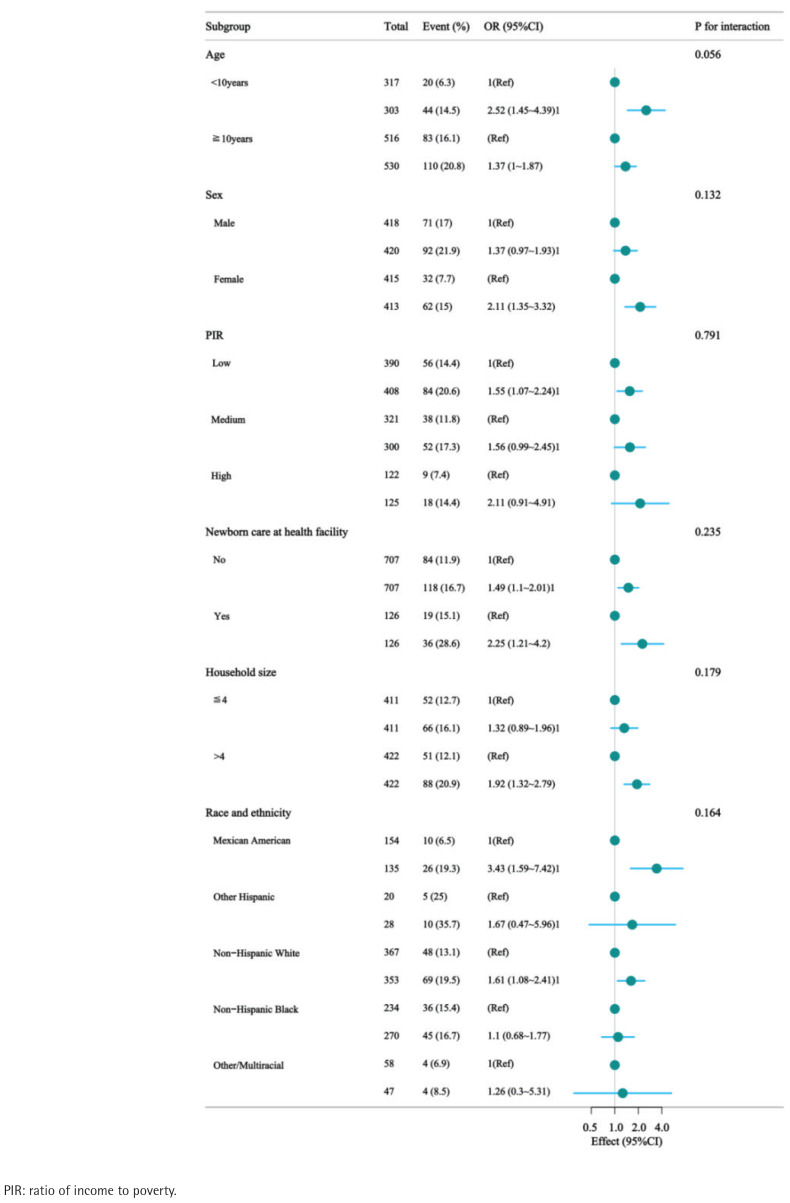
Forest plot of multivariable logistic regression analysis examining the association between mother smoked while pregnant and learning disabilities after matching, National Health and Nutrition Examination Survey (NHANES) cycles 1999–2004

According to subgroup analysis, there were no noteworthy interactions between any of the groupings (Figure 4).

## DISCUSSION

Using NHANES data, this secondary analysis methodically assessed the relationship between maternal smoking during pregnancy and LD in children and adolescents. Using multivariable logistic regression, propensity score matching (PSM), inverse probability weighting (IPW), doubly robust analysis, and stratified analysis, we found a significant positive association between maternal smoking during pregnancy and learning disabilities (LDs) in offspring.

Similar results have also been observed by other investigations. Children whose parents smoked before and during pregnancy were 2.01 times more likely to develop ADHD, according to a Shanghai Children and Adolescents Health Cohort research^12^. Research currently available shows a correlation between prenatal tobacco use and low academic performance, conduct issues, emotional disorders, and ADHD in children^12,20,28^. Maternal smoking during pregnancy roughly triples the risk of learning problems in offspring, according to a large-scale US birth cohort research. In the United States, maternal smoking during pregnancy is directly responsible for 22% of unexpected baby deaths^29^. Additionally, meta-analyses show that prenatal smoking is strongly linked to children’s neurocognitive deficiencies in addition to negative birth outcomes like low birth weight and preterm birth. According to reports, long-term prenatal nicotine exposure alters brain activity during verbal working memory tests, and these effects may last throughout adulthood^11^. Building upon existing evidence, our study employed rigorous methods – including propensity score matching and multiple robustness checks – to reexamine the association between maternal smoking and offspring learning disabilities, thereby providing updated and methodologically strengthened evidence in this critical area.

Substantial evidence indicates that maternal smoking during pregnancy adversely affects fetal neurodevelopment, with several underlying mechanisms proposed to explain these associations. To begin with, the neurotoxic effects of nicotine and carbon monoxide: nicotine can cross the placenta and disrupt neurotransmitter systems in the fetal brain (such as the dopamine and norepinephrine systems), leading to abnormal synaptic development. During early brain development, the cholinergic system also participates in neurite outgrowth, cell survival, proliferation, differentiation, and neurogenesis. The harmful effects of nicotine during these early stages may impact systemic programming and plasticity throughout the individual’s long-term postnatal life^10,30^. Carbon monoxide binds to hemoglobin to form carboxyhemoglobin, causing fetal hypoxia^31^. In addition, prenatal smoking reduces placental blood flow and induces chronic hypoxia, adversely affecting fetal brain development^32^. Lastly, molecular genetic studies demonstrate gene-environment interactions in maternal smoking during pregnancy. Reported genetic variants associated with tobacco smoke metabolite processing (maternal CYP1A1, GSTT1, GSTM1, and norepinephrine transporter gene SLC6A29^13^) may influence polymorphisms in norepinephrine and dopamine transporter genes in offspring, increasing the risk of learning disabilities. Furthermore, epigenetic findings indicate that prenatal smoking alters fetal DNA methylation patterns, potentially exerting long-term effects on learning and cognitive functions^14^.

Smoking during pregnancy is an avoidable risk factor from the standpoint of public health. A successful intervention that targets this behavior may minimize the likelihood of learning difficulties in children as well as the incidence of unfavorable prenatal outcomes.

### Strengths and limitations

The results of this study offer significant epidemiological support for smoking cessation and pregnant health management strategies. To examine the association between maternal smoking during pregnancy and the likelihood of reporting learning disabilities in children and adolescents, we employed robust analytical methods, including multivariable logistic regression, propensity score matching (PSM), doubly robust estimation, inverse probability weighting (IPW), standardized mortality ratio weighting (SMRW), and stratified analyses.

However, it is important to acknowledge the limitations of this research. First, information on pregnant smoking was gathered by questionnaire, which may introduce recall or reporting bias. Second, even with the use of PSM and other robustness techniques, residual confounding factors cannot be completely ruled out due to the cross-sectional nature of NHANES. Third, because the study does not offer detailed information on smoking intensity or cessation time, it is difficult to assess dose-response associations. Finally, the findings from this study may not be generalizable to the broader US population. Randomized controlled trials and prospective cohort studies are needed to further validate this causal relationship and look into potential causes.

## CONCLUSIONS

This study shows a potential association between cognitive deficits (LD) in children and adolescents and maternal smoking during pregnancy. In order to lessen the burden of LD and enhance the long-term health of the unborn child, quitting smoking during pregnancy may be extremely important.

## Data Availability

Publicly available datasets are available online for this study. The repository names and accession numbers are available online at: https://www.cdc.gov/nchs/nhanes/index.htm
